# Compressible and Recyclable Monolithic g-C_3_N_4_/Melamine Sponge: A Facile Ultrasonic-Coating Approach and Enhanced Visible-Light Photocatalytic Activity

**DOI:** 10.3389/fchem.2018.00156

**Published:** 2018-05-18

**Authors:** Ye Yang, Qian Zhang, Ruiyang Zhang, Tao Ran, Wenchao Wan, Ying Zhou

**Affiliations:** ^1^The Center of New Energy Materials and Technology, School of Materials Science and Engineering, Southwest Petroleum University, Chengdu, China; ^2^State Key Laboratory of Oil and Gas Reservoir Geology and Exploitation, Southwest Petroleum University, Chengdu, China

**Keywords:** g-C_*3*_N_*4*_/melamine sponge, photocatalysis, NO removal, monolith, visible light

## Abstract

Powdery photocatalysts seriously restrict their practical application due to the difficult recycle and low photocatalytic activity. In this work, a monolithic g-C_3_N_4_/melamine sponge (g-C_3_N_4_/MS) was successfully fabricated by a cost-effective ultrasonic-coating route, which is easy to achieve the uniform dispersion and firm loading of g-C_3_N_4_ on MS skeleton. The monolithic g-C_3_N_4_/MS entirely inherits the porous structure of MS and results in a larger specific surface area (SSA) than its powdery counterpart. Benefit from this monolithic structure, g-C_3_N_4_/MS gains more exposed active sites, enhanced visible-light absorption and separation of photogenerated carriers, thus achieving noticeable photocatalytic activity on nitric oxide (NO) removal and CO_2_ reduction. Specifically, NO removal ratio is as high as 78.6% which is 4.5 times higher than that of the powdery g-C_3_N_4_, and yield rate of CO and CH_4_ attains 7.48 and 3.93 μmol g^−1^ h^−1^. Importantly, the features of low-density, high porosity, good elasticity, and firmness, not only endow g-C_3_N_4_/MS with flexibility in various environmental applications, but also make it easy to recycle and stable for long-time application. Our work provides a feasible approach to fabricate novel monolithic photocatalysts with large-scale production and application.

## Introduction

Semiconductor photocatalysis is one of the promising strategies for pollutants abatement (Maggos et al., 2007; Huang et al., 2013, 2016; Zhao et al., 2015) and has attracted intense investigation in the past decades. Up to now, hundreds of semiconductors have been explored and applied in the field of environmental remediation (Liu et al., 2008; Hossain and Mukherjee, 2013; Huang et al., 2015a, 2017a; Zhou et al., 2016). However, only a few of them have been considered as potential candidates for practical application in view of their nontoxic, suitable band gap, band edge energy, good stability and earth-abundant source. g-C_3_N_4_ is one of those semiconductors, which possesses graphene-like structure and constituted mainly by carbon and nitrogen. Since g-C_3_N_4_ was first reported to photocatalytic water splitting by Wang et.al (Wang et al., 2009), it quickly becomes a hot material in photocatalysis. Afterwards, g-C_3_N_4_ has already been applied in various reactions such as CO_2_ photoreduction, NO removal, and dye degradation (Yan et al., 2009; Dong et al., 2014a; Sun H. et al., 2017). However, extensive studies revealed that g-C_3_N_4_ suffers from fast photo-generated carriers recombination, limited visible-light absorption, and low surface area. Various strategies have been carried out to overcome these intrinsic drawbacks of g-C_3_N_4_, such as elemental doping, composite with other materials and morphology control synthesis, etc. (Liu et al., 2010; Zhao et al., 2012; Hou et al., 2014; Cheng et al., 2015; Han Q. et al., 2015; Li et al., 2017; Yang et al., 2017). Besides above disadvantages, as a potential photocatalyst for practical application, g-C_3_N_4_ is also hindered by difficult recycle originating from particle heavy loss during its complicated recovery process, inefficient utilization of active sites and light energy resulting from particle aggregation. Comparison with the intrinsic drawbacks of g-C_3_N_4_, these problems are vital to achieve successful application of photocatalyst on actual environmental issues, but they are rarely studied and generally beyond the aforementioned strategies to overcome.

Recently, monolithic or integrated photocatalysts are found to be a plausible way to solve the practical application problem of photocatalyst (Cheng et al., 2016; Wan et al., 2018). The so-called monolithic photocatalyst usually consists of two parts, one is a macroscopical support with porous three-dimensional (3D) skeleton, and the other is the loaded photocatalyst particles. After integrating powdery catalysts on its support, their recycling becomes easy to achieve by a tweezer (Liu W. J. et al., 2015; Tang et al., 2017). Meanwhile, the 3D porous structure of support gives the powdery catalysts a high dispersion and exposes more active sites by avoiding particles agglomeration. Moreover, this structure also benefits light energy harvest and transportation of liquid or gas pollutants. Until now, graphene aerogels are the most studied monolithic photocatalyst supports because of their inherent large surface area, high porosity and low density. We have fabricated monolithic C_3_N_4_/graphene oxide aerogel (GOA) in our previous work and found obvious activity enhancement (Wan et al., 2016), which is in line with the results from other monolithic photocatalyst/graphene aerogels (Fan et al., 2015; Cui et al., 2017; Wang et al., 2017). However, the intrinsic brittleness and weak firmness of aerogels make it easy to break into pieces during mechanical deformation, which seriously restrict its potential in practical application. Other firm supports, such as carbon foam and Al_2_O_3_ ceramic foam with hard 3D framework, are also selected to fabricate monolithic photocatalyst (Dong et al., 2014b; Lin et al., 2016). Nevertheless, it is very difficult to achieve uniform loading by directly mixing photocatalyst with these hard supports. To overcome this problem, special strategies with high cost and energy consumption, like *in-situ* immobilizing approach and laser ablating deposition (Liang et al., 2015; Lin et al., 2016), are used to achieve the good catalyst dispersion. The sophisticated preparation method severely limits the hard supports to be extensively utilized in fabrication of monolithic photocatalyst. Based on the above considerations, the proper support remains an obstacle for the practical application of monolithic photocatalyst. Lately, melamine sponge (MS), a cheap commercial polymer foam which is widely used as kitchen and construction materials, is successfully used for oil-water separation by integrating with graphene (Liu T. T. et al., 2015; Zhao et al., 2016). The fabricated graphene/MS exhibits low-density, high porosity and high elasticity inherited from MS, which exactly match the support characteristics of the monolithic photocatalyst. Importantly, the good elasticity makes MS more ductile and avoids the drawbacks of brittle and hard materials. Therefore, MS is a potential alternative and selected as the support for monolithic photocatalyst fabrication.

Herein, we prepare a monolithic g-C_3_N_4_/MS by a facile ultrasonic-coating method at room temperature, which is very easy to achieve mass production. The monolithic structure endows g-C_3_N_4_/MS with enhanced light harvest and more exposed active sites, ensuring its good photocatalytic activity. Importantly, the as-prepared monolithic photocatalyst exhibits low-density, high porosity, high elasticity and good firmness, which not only make it flexible in various environmental applications including NO removal, and CO_2_ photoreduction, but also make it easy to recycle and suitable for practical application. Overall, our results provide a novel strategy to develop monolithic photocatlyst for practical application with large-scale production.

## Experimental

### Synthesis of g-C_3_N_4_

The polymeric g-C_3_N_4_ was prepared by pyrolysis of urea (Liu et al., 2011). In a typical process, 15 g urea was added into an alumina crucible with a cover and then heated to 550°C in a muffle furnace for 1 h with a heating rate of about 55°C min^−1^. After cool down to room temperature, the final yellow agglomerates were the pristine g-C_3_N_4_ and subsequently ground into powder for further use.

### Preparation of monolithic g-C_3_N_4_/MS

For the preparation of monolithic g-C_3_N_4_/MS, 2.5 g g-C_3_N_4_ powder was dispersed in 500 mL water and sonicated for 1 h to form a g-C_3_N_4_ suspension. MS was cut into suitable size and washed with deionized water and alcohol in order, then dried at room temperature. Next, the clean MS was immersed into g-C_3_N_4_ suspension for 30 min, and then squeezed out the excess solution. After that, the sample was transferred into a culture dish and freeze-dried (−70°C pre-freezing) for 48 h to obtain g-C_3_N_4_/MS. For comparison, powdery g-C_3_N_4_ without MS was processed through the same procedures and denoted as sonicated g-C_3_N_4_. To obtain the best photocatalytic activity of monolithic g-C_3_N_4_/MS, we finely investigated the effect of g-C_3_N_4_ suspension concentration (3 mg mL^−1^-40 mg mL^−1^) and MS thickness (0.5–2.5 cm). The sizes of monolithic g-C_3_N_4_/MS varied with different experiments and were stated at their first appearance in the text.

### Characterization

Powder X-ray diffraction (PXRD) was performed on a PANalytical X'pert diffractometer with a Cu Ka radiation. Transmission electron microscopy (TEM) was performed on a FEI tecnai G2 F30 microscope operated at 200 kV. The morphology of g-C_3_N_4_/MS was observed through scanning electron microscopy (SEM) on a ZEISS EVO MA15 microscopy. The Fourier transform infrared (FT-IR) spectra were measured using a Nicolet 6700 spectrometer on samples embedded in KBr pellets. UV-vis diffuse reflectance spectrum (DRS) data were recorded on a Shimadzu UV-2600 spectrophotometer. Photoluminescence spectra were recorded on F-7000 FL spectrofluorometer with an excitation wavelength at 320 nm. X-ray photoelectron spectroscopy (XPS) was performed by using a Thermo Scientific Escalab 250Xi spectrometer. The specific surface area (SSA) was determined via using methylene blue (MB) adsorption method on a UV-vis spectrophotometer (UV-5100, Anhui Wanyi; Tran et al., 2015), the SSA of g-C_3_N_4_ and g-C_3_N_4_/MS were calculated by the following equation:

$$\begin{array}{l} SSA=\frac{N_{A}A_{MB}\left(C_{0}-C_{e}\right)V}{M_{MB}m_{s}} \end{array}$$

Where *N*_*A*_ represents Avogadro's constant (6.02 × 10^23^ mol^−1^), *A*_*MB*_ represents the covered area of per MB molecule (typically assumed to be 1.35 nm^2^), *C*_*o*_ and *C*_*e*_ are the initial and equilibrium concentrations of MB, *V* is the volume of MB solution, *M*_*MB*_ is the relative molecular mass of MB, and *m*_*s*_ is the mass of the sample.

### Evaluation of photocatalytic activity

The photocatalytic activity of g-C_3_N_4_/MS was evaluated in both gaseous systems. Photocatalytic removal of NO at ppb level was previously reported in details (Zhang et al., 2014). Typically, A 150 W metal halide lamp with a visible light filter (>420 nm) was employed to operate the experiment. A piece of g-C_3_N_4_/MS was put into the reactor for photocatalytic activity test. The light intensity is 35.88 mW cm^−2^ measured by a light intensity meter. The initial concentration of NO was diluted to 500 ppb by drying air. The flow rates of dry air and NO are set at 2 L min^−1^ and 9.5 mL min^−1^, respectively. The formula of degradation rate of NO was counted by the following equation:

$$\omega \left(\%\right)=\frac{[C\left(NO_{X}\right)-C\left(NO\right)-C_{0}\left(NO_{2}\right)}{C\left(NO_{X}\right)}\;\times 100\%$$

Where C (NO_x_) represents the concentration of total nitric oxide (NO_2_ and NO), while C_0_ is the initial concentration of NO_2_ when reaching the adsorption-desorption equilibrium.

The photocatalytic reduction of CO_2_ was performed in a 380 mL home-made reactor at ambient temperature and pressure. A 300 W Xe lamp was used as a light source and positioned 8 cm above the photocatalytic reactor. In a typical test, a plastic beaker with 20 mL deionized water was deposited at the bottom of the reactor, and a culture dish with 100 mg g-C_3_N_4_ powder or g-C_3_N_4_/MS (38.5 cm^2^) was placed on the plastic beaker. Before irradiation, the reactor was sealed and vacuumed by a pump, then removed air by blowing argon for 15 min. Subsequently, 1 mL CO_2_ was injected into the reactor. After 4 h irradiation, 1 mL of gas was taken out from the reactor and analyzed by using a gas chromatograph (Techcomp GC7900) equipped with a flame ionized detector (FID) and a thermal conductivity detector (TCD). CO and CH_4_ were analyzed by the FID, and H_2_ was analyzed by TCD.

## Results and discussion

### Fabrication and physical properties of g-C_3_N_4_/MS

**Figure 1:**
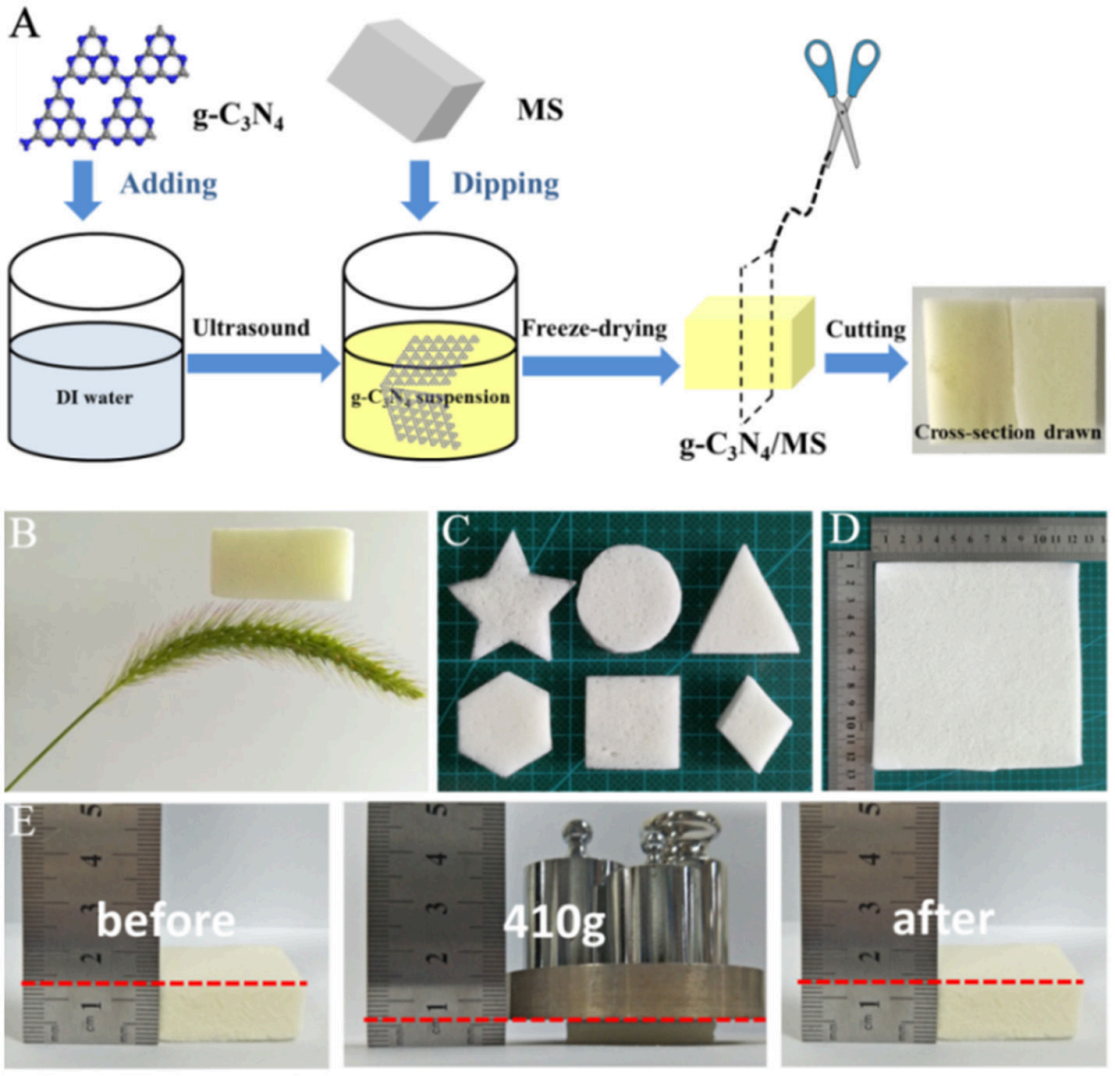
**(A)** Preparation process of g-C_3_N_4_/MS photocatalyst; **(B)** Ultra-light g-C_3_N_4_/MS resting on dog tail grass; **(C)** g-C_3_N_4_/MS with various shapes; **(D)** g-C_3_N_4_/MS with large area (12.0 × 12.0 × 0.5 cm^3^); **(E)** Mechanical property of g-C_3_N_4_/MS.

The general preparation approach of g-C_3_N_4_/MS was illustrated in Figure 1A. Direct mixture of powdery g-C_3_N_4_ and MS is hard to gain a monolithic g-C_3_N_4_/MS with good particle dispersion and well contact between particle and MS skeleton. Therefore, the g-C_3_N_4_ powder was firstly added into water and sonicated for 30 min to form uniform suspension, which make the particle diffusion easy and fast in the porous structure of MS. Then, the pretreated MS was immersed into this suspension through dipping and squeezing procedures until MS was fully covered by g-C_3_N_4_ particles. Finally, followed by a conventional freezing-drying process, the monolithic g-C_3_N_4_/MS was obtained. Obviously, no large particles were observed on the cross-section photo in Figure 1A, indicating that the g-C_3_N_4_ powder was uniformly coated on MS skeleton. For comparison, we also dipped MS in a saturated solution of urea, which is the g-C_3_N_4_ precursor. After removing the water by freeze-drying, we attempted to fabricate the monolithic g-C_3_N_4_/MS by *in-situ* immobilizing approach at 550°C. However, the obtained monolithic composite became very fragile due to the carbonization of MS skeleton. Moreover, the g-C_3_N_4_ powder gathered on the composite surface with an inhomogeneous dispersion (Figure S1). The failed trial illustrated the mild ultrasonic-coating approach is superior to other methods for monolithic g-C_3_N_4_/MS fabrication. Importantly, the facile ultrasonic-coating approach developed here is also adapted to other porous supports, such as nickel foam, Al_2_O_3_ ceramic foam, glass fiber and polyester fiber, and the corresponding monolithic products of g-C_3_N_4_ are shown in Figure S2.

Physical properties of materials are pivotal to their practical application. As shown in Figure 1B, the monolithic g-C_3_N_4_/MS has an ultra-low density (11.5 mg cm^−3^), which can stay on dog tail grass and remarkably lighter than that of reported BiOBr/reduced GOA (50 mg cm^−3^) (Liu W. J. et al., 2015), TiO_2_/graphene aerogel (19 mg cm^−3^) (Qiu et al., 2014), and MoS_2_/reduced GOA (56.1 mg cm^−3^) (Zhang R. Y. et al., 2017). Moreover, SSA of g-C_3_N_4_/MS (7.6 m^2^ g^−1^) is much more larger than that of the pristine g-C_3_N_4_ (0.9 m^2^ g^−1^), which not only offer more active sites but also have larger absorption capacity than its powdery counterpart. In addition, facile modification of shapes and sizes endows g-C_3_N_4_/MS with a good flexibility to handle different situations in practical application (Figures 1C,D). More importantly, the g-C_3_N_4_/MS presents excellent elasticity. As revealed in Figure 1E, the g-C_3_N_4_/MS can instantaneously recover and maintain its integrity after removing of the heavy loading (410 g counterweight), suggesting that g-C_3_N_4_/MS possesses enough mechanical strength to deal with intricate operation in environmental abatement. To further ensure the firmness of g-C_3_N_4_ on MS skeletons, a test is carried out by blowing the g-C_3_N_4_/MS with a strong airflow for 12 h, the detailed simulation device diagram is shown in Figure S3a. The dropping g-C_3_N_4_ powder from g-C_3_N_4_/MS is collected and weighted up. The final weight loss is less than 7.60 mg, which account for 1.15% of g-C_3_N_4_ loaded on MS skeletons (Figure S3b). The above result demonstrates that g-C_3_N_4_ is firmly distributed on MS even under extreme work condition, which is pivotal to the recycle in practical application.

### Photocatalytic activity

**Figure 2:**
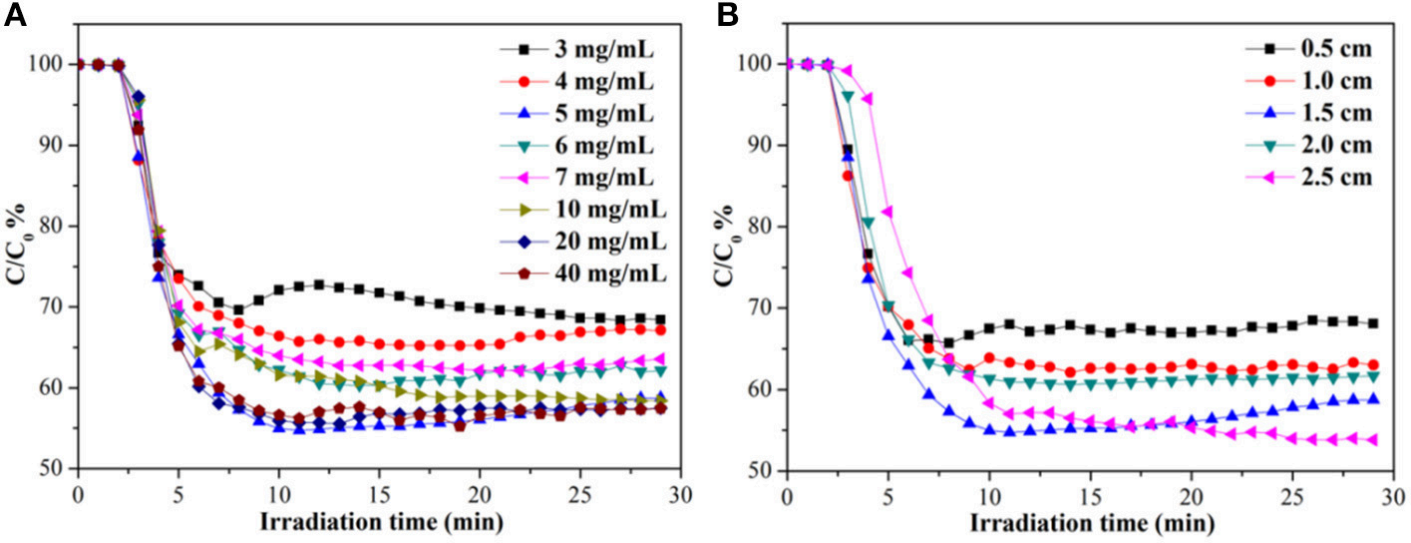
Photocatalytic NO removal ratios in presence of g-C_3_N_4_/MS fabricated with **(A)** different concentrations and **(B)** different thicknesses under visible light irradiation.

Benefit from the characteristics of low-density, high porosity and good elasticity, the monolithic g-C_3_N_4_/MS can be used to removal gaseous pollutants. Therefore, two different photocatalysis applications including NO removal, dye degradation, and CO_2_ photoreduction, are selected to test the photocatalytic activity of the as-prepared monolithic g-C_3_N_4_/MS. NO, a typical air contaminants, mostly produced from the combustion of fossil fuels and the emission of vehicle exhaust, can cause a series of atmosphere pollution problems such as acid rain, photochemical smog and haze (Wang et al., 2016). Photo-oxidation technique is considered an alternative to remove NO at low concentration (Zhou et al., 2014). Therefore, the g-C_3_N_4_/MS samples are firstly investigated by NO removal at the indoor ppb level. Figure 2A shows the effect of g-C_3_N_4_ concentration on NO photo-removal ratio occurring on g-C_3_N_4_/MS. Notably, the MS coated with 5 mg mL^−1^ suspension achieves the highest removal ratio of 45% within 30 min. As the concentration of g-C_3_N_4_ suspension less than 5 mg mL^−1^, the photocatalytic activity of g-C_3_N_4_/MS gradually enhanced with the increased concentration of g-C_3_N_4_ suspension. With a concentration higher than 5 mg mL^−1^, the photocatalytic activity of the g-C_3_N_4_/MS slightly decreased, attributing to agglomeration of the excessive g-C_3_N_4_ which not only hinder the NO transport by blocking pore channels in MS, but also cause a reduced light transmittance. Moreover, the thicknesses of MS have also been investigated, which is closely associated with the light utilization. Benefit from the good transparence of MS, the activity of g-C_3_N_4_/MS enhanced along with increased MS thickness in Figure 2B, but the corresponding best unit mass rate constant (0.868 min^−1^ g^−1^) of NO removal is belong to g-C_3_N_4_/MS with 0.5 cm thickness (As shown in Figure S4). Based on above results, the optimal concentration and thickness were fixed at 5 mg mL^−1^ and 0.5 cm, respectively.

**Figure 3:**
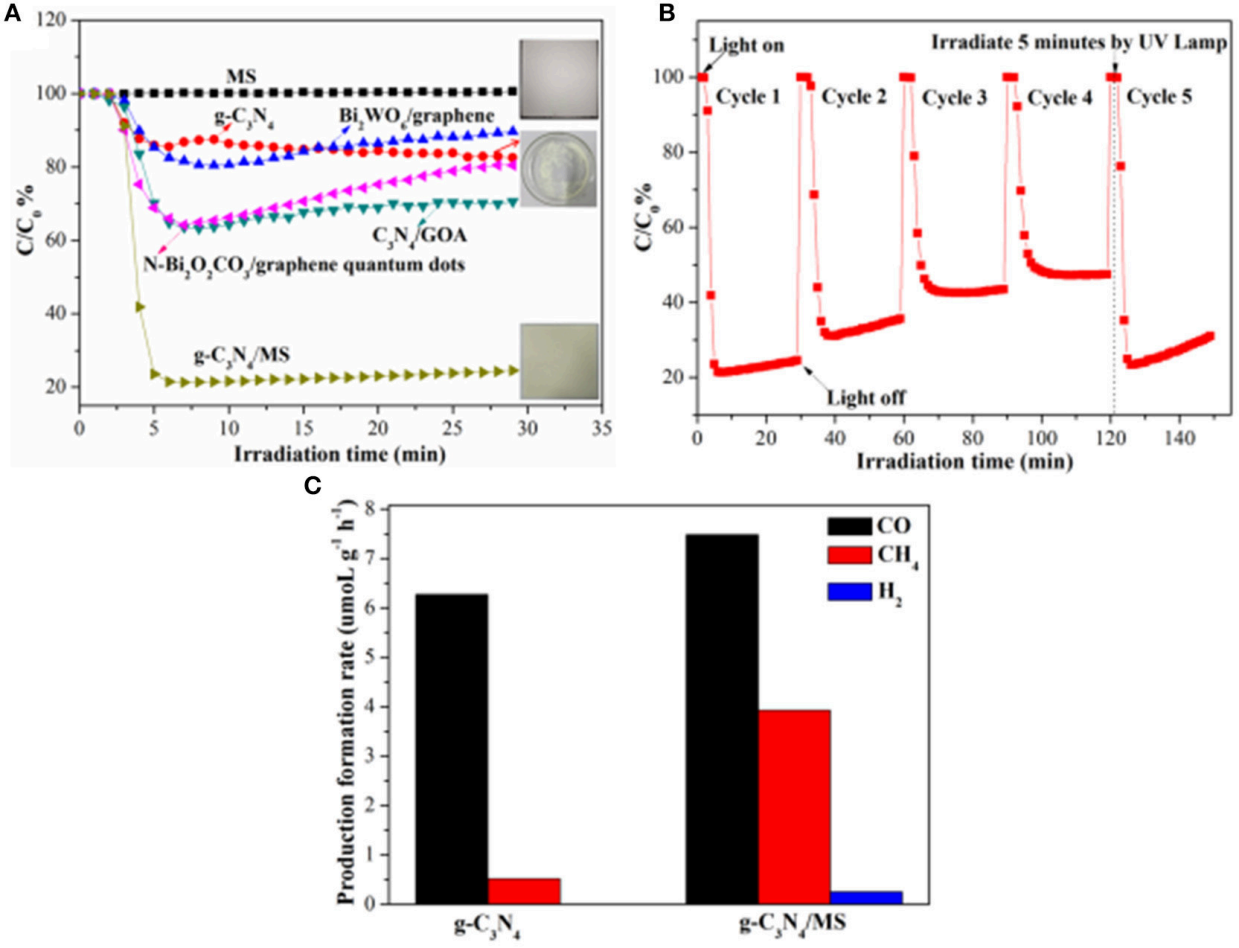
Photocatalytic activity of g-C_3_N_4_/MS under visible-light irradiation: **(A)** NO removal ratios of different samples; **(B)** Photocatalytic recycling test on large area g-C_3_N_4_/MS; **(C)** Production rate of CO, CH_4_ and H_2_ on g-C_3_N_4_, g-C_3_N_4_/MS respectively in photocatalytic CO_2_ reduction under UV-Vis light irradiation.

The fabrication and utilization of monolithic photocatalysts with large area on macro-scale is significant to their practical application. Therefore, a monolithic g-C_3_N_4_/MS with area of 12.0 × 12.0 cm^2^ is prepared for further test of NO removal under visible-light illumination (Figure 3A). For comparison, the NO removal on powdery g-C_3_N_4_ and pristine MS are also performed under the same conditions, respectively. Surprisingly, monolithic g-C_3_N_4_/MS presents the highest NO removal ratio of 78.6% in initial 5 min which is about 4.5 times higher than that of powdery g-C_3_N_4_ (17.6%), while no NO removal occurs on the pristine MS. After initial 5 min, NO removal ratio of g-C_3_N_4_/MS tends to be steady, which could be interpreted as partial active sites replaced by adsorptive ${\text{NO}}_{3}^{-}$ or NO (Ai et al., 2009; Huang et al., 2010; Liu et al., 2017) and finally reached an adsorption and desorption equilibrium of these oxynitrides. Moreover, comparison with other photocatalysts in our previous work, large area g-C_3_N_4_/MS exhibits optimum activity (78.6%), which is approximate 4.25, 2.25 and 2.32 times higher than that of Bi_2_WO_6_/graphene (Zhou et al., 2014), C_3_N_4_/GOA (GOA) (Wan et al., 2016), and N-Bi_2_O_2_CO_3_/graphene quantum dots (Liu et al., 2017), respectively. Notably, the fraction of generated NO_2_ is lower than 5.4% over all our samples (as shown in Figure S5a), indicating those samples selectively oxidize NO to ${\text{NO}}_{3}^{-}$ rather than NO_2_. Based on above results, the g-C_3_N_4_/MS with large area shows obviously enhanced photocatalytic activity than its powdery counterpart, indicating the monolithic photocatalyst is an effective strategy to solve the problems encountered by powdery photocatalysts in large-scale application.

The stability and recyclability of photocatalysts are also important to their practical applications (Hu et al., 2017). Here, we carried out cycle and long-time tests to further evaluate the performance of the monolithic g-C_3_N_4_/MS. The result of cycle test is listed in Figure 3B. It can be seen that the NO removal ratio of g-C_3_N_4_/MS dropped quickly during the first two cycles. After that, the NO removal ratio approached to stabilization within third cycle and slightly decreased with incremental cycle-index, which attributed to temporary absorption equilibrium of oxynitrides (NO, ${\text{NO}}_{3}^{-}$) in third cycle and their continuous accumulation on g-C_3_N_4_/MS. Fortunately, after UV lamp irradiation for 5 min, the adsorbed oxynitrides are desorbed and results in an immediate recovery of sample activity (Figure 3B). Furthermore, result of long-time test is displayed in Figure S5b. The g-C_3_N_4_/MS can achieve a stable catalytic performance after 1 h and keep a relatively high activity in the 2 h test interval. All above results confirm that the large area g-C_3_N_4_/MS has a superior recyclability and stability for NO removal, confirming its potential in practical application.

Apart from the photo-oxidation availability, the monolithic g-C_3_N_4_/MS also exhibits good ability of CO_2_ photoreduction. CO_2_ is the main greenhouse gas that generation from human activity and the combustion of fossil fuels which is responsible for global warming (Norby and Luo, 2004). As displayed in Figure 3C, the g-C_3_N_4_/MS shows higher photocatalytic activity (7.48 μmol g^−1^ h^−1^ CO, 3.93 μmol g^−1^ h^−1^ CH_4_ and 0.26 μmol g^−1^ h^−1^ H_2_) than that of powdery g-C_3_N_4_ (6.27 μmol g^−1^ h^−1^ CO, 0.52 μmol g^−1^ h^−1^ CH_4_ and 0 μmol g^−1^ h^−1^ H_2_). Notably, yield of CO is higher than that of CH_4_ on both two samples, because the conversion from CO_2_ to CO is 4-electron process, whereas that from CO_2_ to CH_4_ is 8-electrons process, obviously, the former is easier than the latter which can account for higher yield of CO. No H_2_ generated on g-C_3_N_4_ and traced H_2_ appeared on g-C_3_N_4_/MS indicate that all samples have high selectivity for CO_2_ reduction rather than H_2_ reduction.

In view of above results of NO removal, and CO_2_ photoreduction, the monolithic g-C_3_N_4_/MS does show practical potential in various applications and enhanced photocatalytic activity than its powdery counterpart. The large SAA must be responsible for this activity enhancement. However, to get a deep insight into the reasons of the improved performance, more investigations further carried out on the monolithic g-C_3_N_4_/MS.

### Structure and morphology

**Figure 4:**
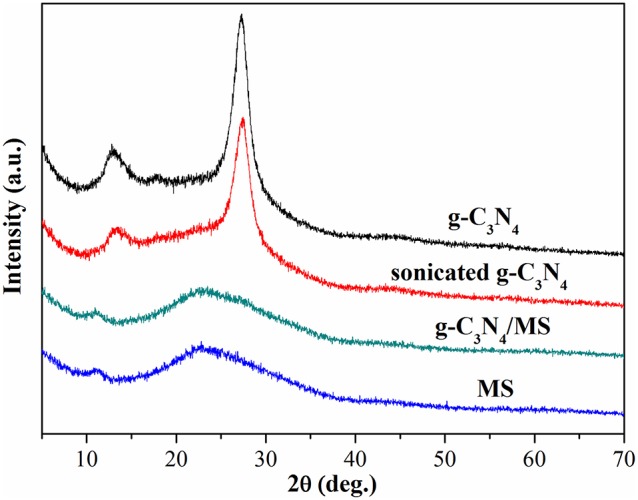
PXRD patterns of g-C_3_N_4_, sonicated g-C_3_N_4_, g-C_3_N_4_/MS and MS.

The PXRD patterns of MS, g-C_3_N_4_/MS, sonicated g-C_3_N_4_ and g-C_3_N_4_ are displayed in Figure 4. The peaks at ca. 13.1 and 27.3° can be assigned to (100) and (002) crystal planes of g-C_3_N_4_, respectively (Gholipour et al., 2016). Obviously, the PXRD patterns of g-C_3_N_4_ are consistent before and after ultrasonic process, confirming the crystal structure of g-C_3_N_4_ is very stable. Moreover, no difference is found in the PXRD patterns of g-C_3_N_4_/MS and MS, indicating the g-C_3_N_4_ particles are uniformly dispersed on the porous framework of MS rather than aggregated on its surface.

**Figure 5:**
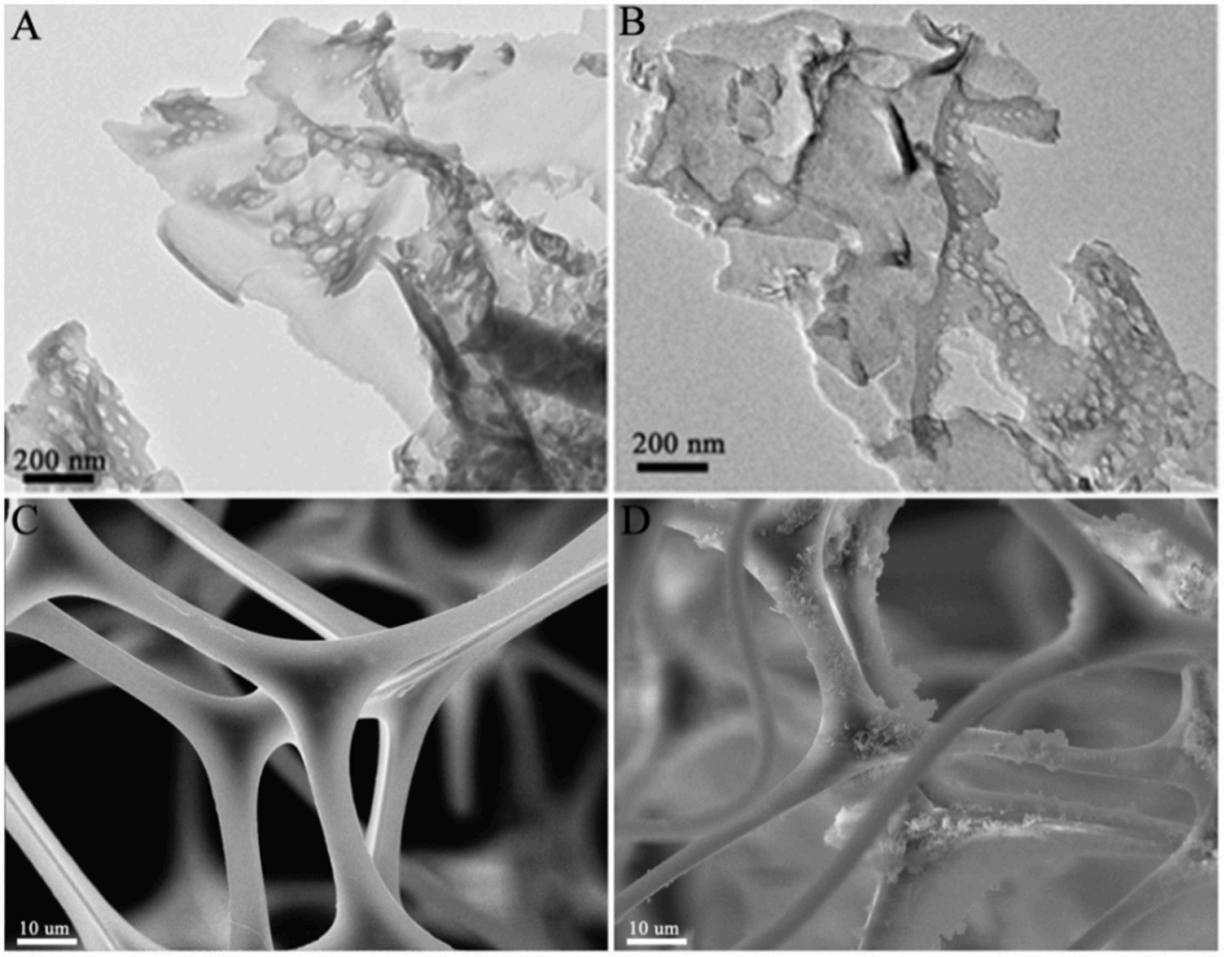
TEM images of samples: **(A)** g-C_3_N_4_; **(B)** sonicated g-C_3_N_4_. SEM images of samples: **(C)** MS; **(D)** g-C_3_N_4_/MS.

To reveal the microstructure and morphology of samples, TEM and SEM were conducted as shown in Figure 5. The TEM images of g-C_3_N_4_ and sonicated g-C_3_N_4_ show similar thin nano-flake structure with some mesopores (Figures 5A,B), which derived from gases releasing such as NH_3_ and CO_2_ during the pyrolysis of urea (Mao et al., 2013). Combination with PXRD results, it is sure no noticeable change appeared on the structure and morphology of g-C_3_N_4_ before and after the ultrasonic. In Figure 5C, the SEM image of MS reveals an interconnected 3D network structure with abundant open-cell pores, which not only offer sufficient channels for reactant transport, but also offer enough locations for photocatalyst particle dispersion. It is notable that the g-C_3_N_4_ was successfully coated on the smooth skeleton of MS according to the g-C_3_N_4_/MS image in Figure 5D. Based on the analysis of structure and morphology, the uniform dispersion of g-C_3_N_4_ achieves in monolithic g-C_3_N_4_/MS, confirming the feasibility of our coating strategy.

**Figure 6:**
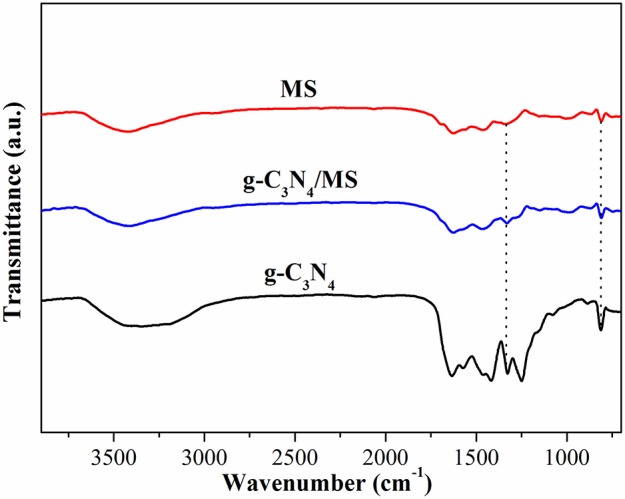
FTIR spectra of g-C3N4, g-C3N4/MS and MS.

Figure 6 shows the FT-IR spectra of g-C_3_N_4_, g-C_3_N_4_/MS and MS. The spectrum of g-C_3_N_4_ displays typical peaks at 3000-3600 cm^−1^, 1200-1700 cm^−1^, and 811 cm^−1^, which are ascribed to the vibrational absorption of N-H and O-H, CN heterocycles and triazine unit (Kang et al., 2015; Wei et al., 2016; Sun Z. X. et al., 2017). Moreover, in the spectrum of MS, the prominent peaks located at 808, 1154, 1545, and 3422 cm^−1^, attributing to triazine ring bending, C-O stretching, C = N stretching and N-H stretching, while peaks centered at 988, 1329, and 1466 cm^−1^ corresponding to C-H bending vibrations (Pham and Dickerson, 2014; Zhang W. B. et al., 2017). Particularly, a new peak and an intensive peak appeared at 1334 cm^−1^ and 813 cm^−1^ in g-C_3_N_4_/MS spectrum, indicating a weak chemical interaction exists between g-C_3_N_4_ and MS skeletons. The above results reveal both van der waals and chemical interactions between g-C_3_N_4_ and MS, which explain the good firmness of monolithic g-C_3_N_4_/MS.

### Band structure and photocatalytic mechanism

**Figure 7:**
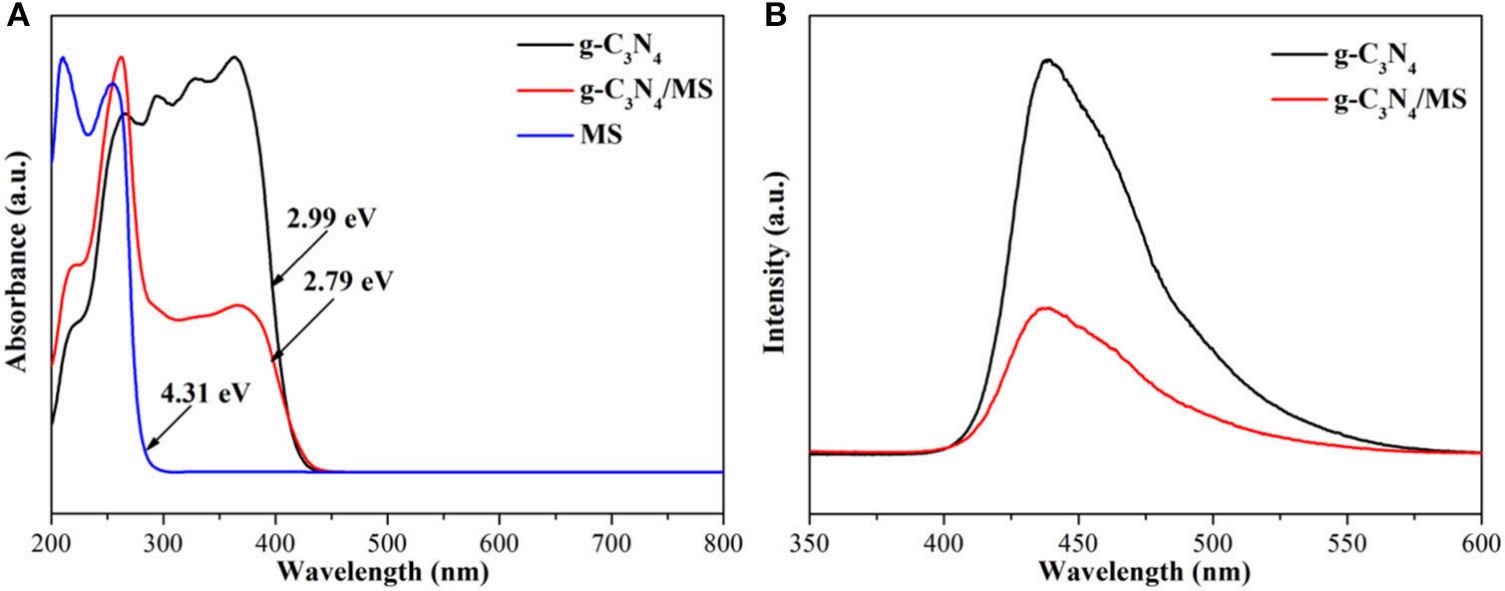
**(A)** UV-vis DRS of g-C3N4, g-C3N4/MS and MS; **(B)** PL spectra of g-C3N4, g-C3N4/MS.

The band gap of MS, g-C_3_N_4_ and g-C_3_N_4_/MS were determined by the results of UV-vis DRS in Figure 7A. Obviously, g-C_3_N_4_ absorption is located in visible region with a calculated band gap of 2.99 eV, while MS shows only UV light absorption with a wide band gap of 4.31 eV. Moreover, the DRS profile of the g-C_3_N_4_/MS exhibits a mechanical combination of the absorption features of the g-C_3_N_4_ and MS alone. Importantly, visible-light absorption of g-C_3_N_4_/MS gets a slight enhancement (2.79 eV), which may be attributed to the light multistage refraction and reflection on the MS framework (Dong et al., 2014b). In addition, the g-C_3_N_4_/MS not only enhances the light absorption, but also significantly suppresses the recombination of photo-generated carriers according to photoluminescence (PL) spectra in Figure 7B, which accounts for the enhancement of photocatalytic activity.

**Figure 8:**
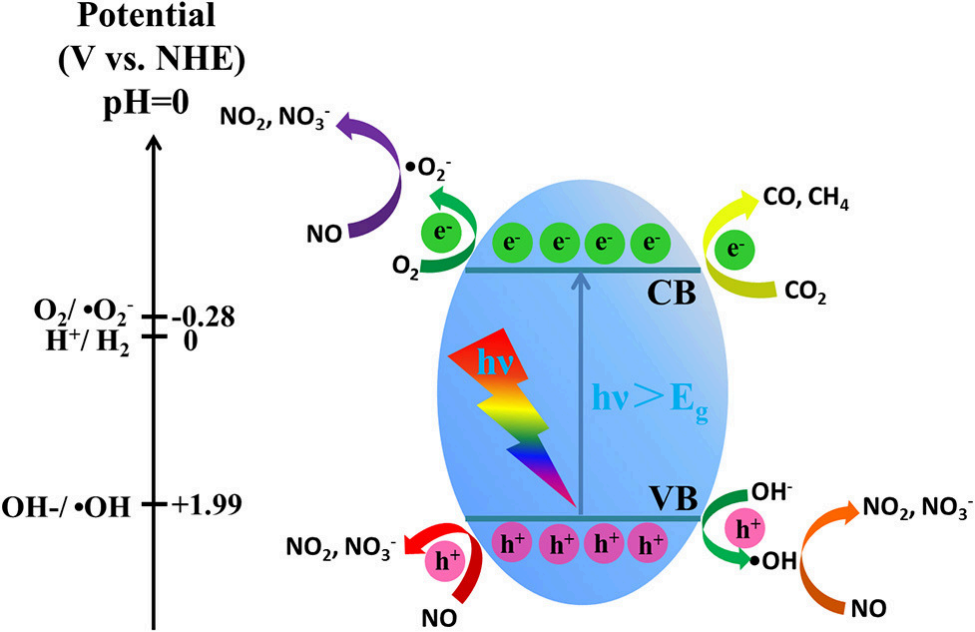
Schematic illustration of the photocatalytic process over the g-C3N4/MS.

According to the above UV-vis DRS analysis (Figure 7A) and the XPS valence band spectrum (Figure S6), the band structure of g-C_3_N_4_/MS is proposed in Figure 8 with VB edge and CB edge located at 2.01 and −0.98 eV, respectively. As shown in Figure 8, the potential of VB holes (h^+^) is slight positive than OH^−^/OH (1.99 eV), while the potential of CB electron (e^−^) is much negative than that of O_2_/${\text{O}}_{2}^{-}$ (−0.28 eV). Therefore, the photogenerated holes can directly oxidize OH^−^ to OH, and the photogenerated electrons can reduce easily O_2_ to ${\text{O}}_{2}^{-}$. It is well known that OH and ${\text{O}}_{2}^{-}$ usually have strong oxidative ability and play the key role in photocatalytic oxidation reaction (Huang et al., 2015b,c, 2017b). Moreover, in consideration of the oxidation potentials of NO_2_/NO (1.03 eV), HNO_2_/NO (0.99 eV), HNO_3_/NO (0.94 eV) (Wan et al., 2016), all OH, ${\text{O}}_{2}^{-}$ and hole generated on g-C_3_N_4_/MS are able to remove NO. In addition, the reduction potentials of E (CO_2_/CH_4_), E (CO_2_/CO) and E (H_2_O/H_2_) were located at −0.24, −0.52, and −0.41 eV, respectively (Yu et al., 2014; Han B. et al., 2015). Comparison with CB potential (−0.98 eV), the photogenerated electron is capable of reducing CO_2_ on g-C_3_N_4_/MS. Based on above analyses and the results in photocatalytic activity part, the possible photocatalytic mechanism are simply illustrated in Figure 8. In short, there are three main pathways for NO removal, involving three different active oxidation species (OH, ${\text{O}}_{2}^{-}$ and h^+^), while there is one pathway for CO_2_ photoreduction, involving only one active species (e^−^).

## Conclusions

In summary, a novel monolithic g-C_3_N_4_/MS was fabricated by a facile ultrasonic-coating method. This monolithic g-C_3_N_4_/MS possesses a uniform dispersion of g-C_3_N_4_ and large SSA, which not only facilitate exposing more active sites of g-C_3_N_4_ but also enhancing the visible-light absorption. Consequently, monolithic g-C_3_N_4_/MS shows obviously improved visible-light photocatalytic activity. The PL detection further reveals that enhanced separation of photogenerated carriers is also responsible for the activity enhancement. More importantly, the characteristics of low-density and high porosity allow the monolithic g-C_3_N_4_/MS to be applied in various environmental issues, while the high elasticity, good firmness, and mechanical strength give it noticeable recyclability and stability, confirming its feasibility for practical application. All in all, in this work, we fabricated a monolithic g-C_3_N_4_/MS with enhanced photocatalytic activity which can be easy to achieve large-scale production for practical photocatalysis application. Our results provide a low-cost and mild method for mass production of new monolithic photocatalysts.

## Author contributions

YY: Partly designed the experiments and wrote the manuscript; RZ, TR, and WW: Assisted in the analysis and interpretation of the data; YZ and QZ: Proposed the project, designed the experiment, and wrote the manuscript.

### Conflict of interest statement

The authors declare that the research was conducted in the absence of any commercial or financial relationships that could be construed as a potential conflict of interest.
