# Supplementary material for: Compressible and Recyclable Monolithic g-C3N4/Melamine Sponge: A Facile Ultrasonic-Coating Approach and Enhanced Visible-Light Photocatalytic Activity
Source: Front Chem. 2018 May 18;6:156. doi: 10.3389/fchem.2018.00156 (PMC5968098; doi:10.3389/fchem.2018.00156)
Supplement: Supplementary file 1 [file Presentation_1.pdf]

Supplementary Material

Compressible and recyclable monolithic g-C_3N_4/melamine sponge: A facile ultrasonic-coating approach and enhanced visible-light photocatalytic activity

Ye Yang[1,2], Qian Zhang[1*], Ruiyang Zhang[1], Tao Ran[1], Wenchao Wan[1], Ying Zhou[1,2*]

[1]The Center of New Energy Materials and Technology, School of Materials Science and Engineering, Southwest Petroleum University, Chengdu, China

[2]State Key Laboratory of Oil and Gas Reservoir Geology and Exploitation, Southwest Petroleum University, Chengdu, China
Fax: +86 28 83037406; Tel: +86 28 83037411

***Correspondence:** Qian Zhang: zhangqian@swpu.edu.cn;

Ying Zhou: yzhou@swpu.edu.cn

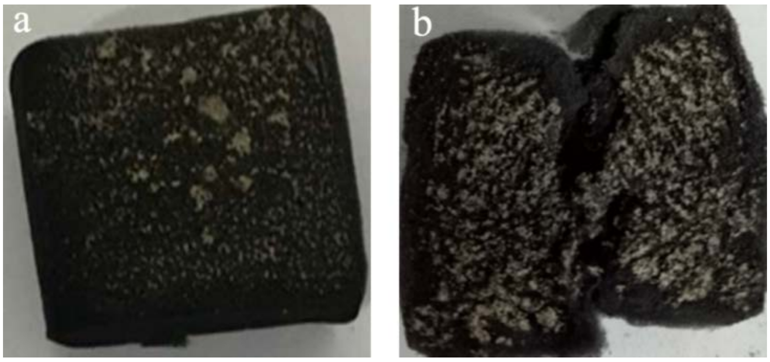

Figure S1 | (a) Photographs of melamine sponge soaked in saturated urea solution through high temperature calcining treatment; (b) the corresponding section drawing.

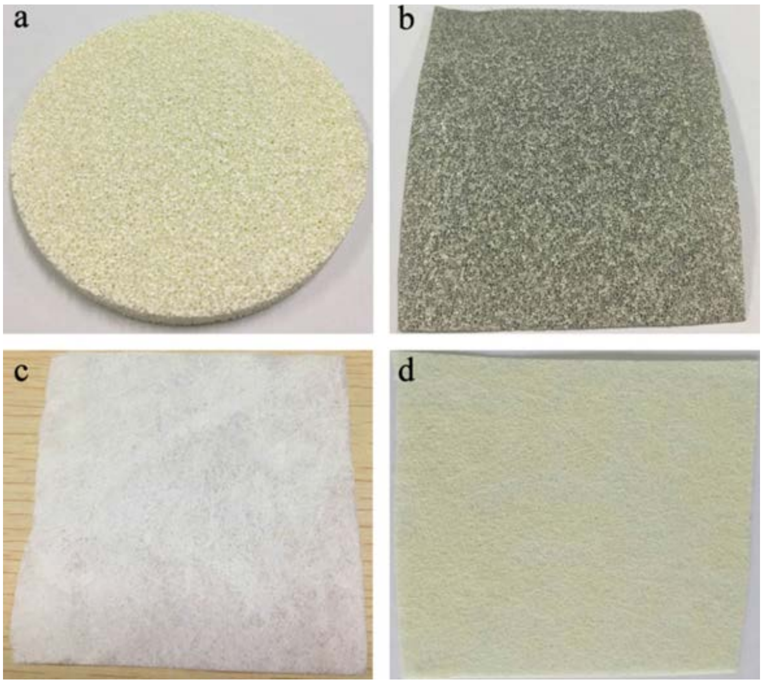

Figure S2 | Photographs of monolith photocatalysts (a) g-C_3N_4/Al_2O_3 ceramic foam; (b) g-C_3N_4/ nickel foam; (c) g-C_3N_4/glass fibre; (d) g-C_3N_4/polyester fibre.

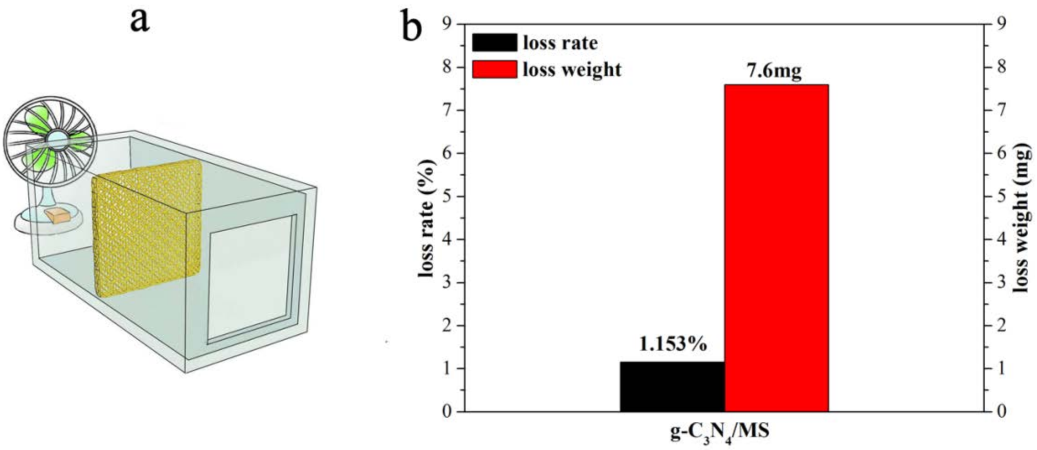

Figure S3 | (a) The simulation binding force test experimental setup of large area g-C_3N_4/MS sample (the distance between sample and fan is 15 cm); (b) the corresponding weight loss and loss rate (the weight of loss/ catalyst loading × 100%) of the supported samples.

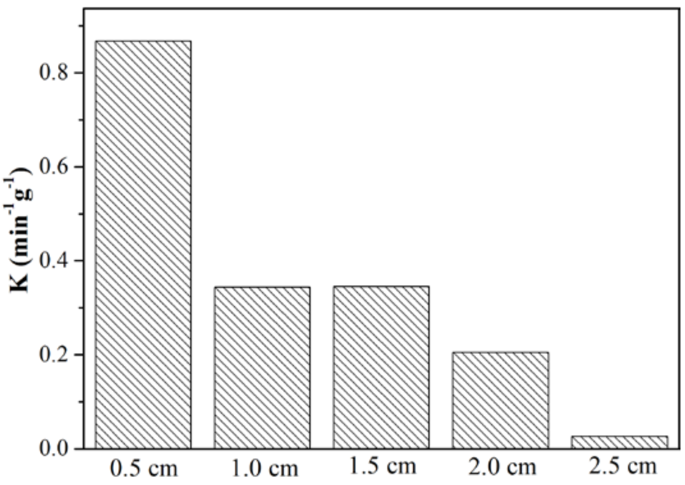

Figure S4 | The unit mass Arrhenius rate constants of different thicknesses g-C_3N_4/MS samples for the removal of NO under visible light irradiation.

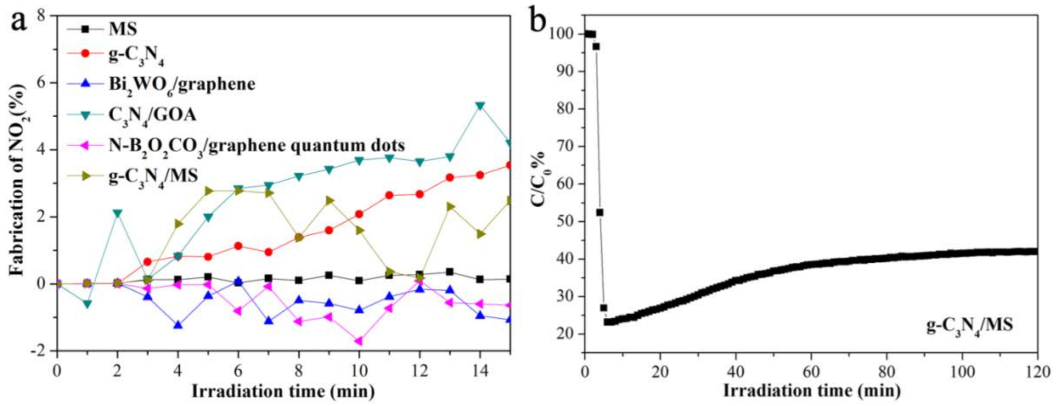

Figure S5 | (a) Monitoring of NO_2 intermediates over the large area g-C_3N_4/MS under visible light irradiation; (b) The photodegradation of NO over the large area g-C_3N_4/MS in visible light vs prolonged irradiation time.

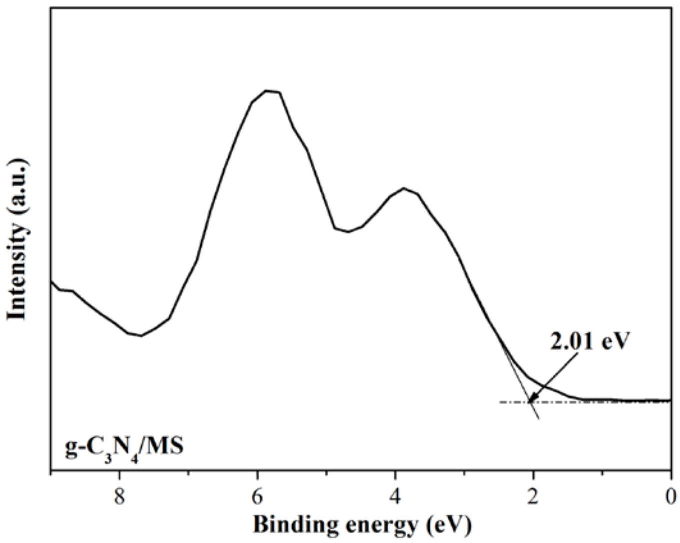

Figure S6 | XPS valence band spectra of g-C_3N_4/MS.
